# The application of ultrasound in detecting lymph nodal recurrence in the treated neck of head and neck cancer patients

**DOI:** 10.1038/s41598-017-04039-3

**Published:** 2017-06-21

**Authors:** Chi-Maw Lin, Cheng-Ping Wang, Chun-Nan Chen, Che-Yi Lin, Ting-Yi Li, Chen-Han Chou, Ya-Ching Hsu, Po-Yen Kuo, Tsung-Lin Yang, Pei-Jen Lou, Jenq-Yuh Ko, Tseng-Cheng Chen

**Affiliations:** 1 Department of Otolaryngology, National Taiwan University Hospital, Yun-Lin Branch, Yun-Lin, Taiwan; 2 Department of Otolaryngology, National Taiwan University Hospital and National Taiwan University, College of Medicine, Taipei, Taiwan; 3 Department of Otolaryngology, National Taiwan University Hospital, Hsin-Chu Branch, Hsin-Chu, Taiwan; 4 Department of Pathology, National Taiwan University Hospital, Yun-Lin Branch, Yun-Lin, Taiwan

## Abstract

Early detection of neck lymph node (LN) recurrence is paramount in improving the prognosis of treated head and neck cancer patients. Ultrasound (US) with US-guided fine needle aspiration (FNA) and core needle biopsy (CNB) have been shown to have great accuracy for LN diagnoses in the untreated neck. However, in the treated neck with fibrosis, their roles are not clarified. Here, we retrospectively review 153 treated head and neck cancer patients who had received US and US-guided FNA/CNB. In multivariate logistic regression analyses, size (short-axis diameter >0.8 cm) (odds ratio (OR) 4.19, P = 0.007), round shape (short/long axis ratio >0.5) (OR 3.44, P = 0.03), heterogeneous internal echo (OR 3.92, P = 0.009) and irregular margin (OR 7.32, P < 0.001) are effective US features in predicting recurrent LNs in the treated neck. However, hypoechogenicity (OR 2.38, P = 0.289) and chaotic/absent vascular pattern (OR 3.04, P = 0.33) are ineffective. US-guided FNA (sensitivity/specificity: 95.24%/97.92%) is effective in the treated neck, though with high non-diagnostic rate (29.69%). US-guided CNB (sensitivity/specificity: 84.62%/100%) is also effective, though with low negative predictive value (62.5%). Overall, US with US-guided FNA/CNB are still effective diagnostic tools for neck nodal recurrence surveillance.

## Introduction

Among all recurrent head and neck cancers, recurrences in the lymph nodes (LNs) of the neck are the most common, with an incidence of approximately 31–42%^1^. Therefore, early detection of neck nodal recurrence is paramount in improving the success rates of salvage treatment and the final prognosis^2^. However, nearly all previous treatments will increase the difficulties in detecting LN recurrence in the treated neck of patients with head and neck cancers. The preceding radiation will lead to fibrotic tissue changes in the treated neck^3^. Preceding surgery such as neck dissection or flap reconstruction will cause anatomic variations in the treated neck. The tissue repair process after surgery also leads to fibrotic tissue changes and scar formation. All of these changes will increase the difficulties in detecting LN recurrence in the treated neck. Consequently, it is sometimes difficult to exactly confirm LN recurrence by means of neck palpation, even with an experienced clinician. Therefore, in routine practice, we frequently rely on several types of image examinations, such as neck ultrasound (US), computed tomography (CT), magnetic resonance imaging (MRI) and positron emission tomography (PET) to detect possible LN recurrence in the treated neck.

Compared to CT, MRI and PET, US examination has several advantages. First, it does not have the risk of radiation overexposure, even after several examinations. Second, it is more acceptable by head and neck cancer patients because it is less expensive and the necessary examination time is shorter than that of CT, MRI and PET. Most importantly, when there are suspicions of recurrent cancer in the LNs during the examination, the clinician can perform ancillary tissue sampling using US-guided fine needle aspiration (FNA) or core needle biopsy (CNB) simultaneously. With the histologic verification of cell cytology and/or tissue pathology, the clinician can more confidently make appropriate decisions regarding management.

Regarding the practice of US surveillance of the treated neck, there are questions that need to be answered. First, the detection of malignant LNs mainly depends on US features such as size (short-axis diameter >0.8 cm), hypoechogenicity (with respect to the surrounding muscles, except hyperechoic hilum), heterogeneous type of internal echo (coarse or inconsistent LN texture, expect hyperechoic hilum), irregular margin (interrupt, ill-defined, or unsharp margin), chaotic/absent vascular pattern (loss of hilum blood flow or increased peripheral blood flow) and round shape (taller than wide, short/long axis ratio >0.5)^4–9^. However, the impact of these malignant US features mainly comes from the series focusing on the untreated neck so that the effect of previous radiation and/or surgery on the soft tissue and LNs in the treated neck cannot be clarified. Does the preceding treatment change the expression of suspicious US features in the treated neck? Second, the ancillary US-guided FNA/CNB have been shown to have great accuracy in replacing open excisional biopsy in the untreated neck^10–14^. For the treated neck, which has more scar tissue and anatomic changes after previous surgery or radiotherapy, open excisional biopsy is further associated with increased risk of complications such as large vessel injuries and poor wound healing. Are the US-guided FNA/CNB still good enough in the treated neck to provide accurate histologic verification and to replace open excisional biopsy?

The aims of our study are to analyze the impact of different US features in detecting LN recurrence and to clarify the role of US-guided FNA/CNB in the histologic verification of LN recurrence in the treated neck.

## Results

### Patient demographics

There were 219 treated head and neck cancer patients who had received US exam after primary curative treatment in our hospital from January 2011 to December 2014. After excluding the patients who did not receive FNA or CNB and the patients who did not have complete medical and image records, a total of 153 eligible patients were enrolled in this study, including 134 male and 19 female patients. Their ages ranged from 22 to 76 years, with a mean age of 52 ± 9 years. Of these 153 patients, 68 patients had a definite diagnosis of LN recurrence in the treated neck. Concerning the previous neck treatment, 67 patients received radiation alone, 50 patients received surgical neck dissection alone, and 36 patients received both surgical neck dissection and radiation. The clinical characteristics of all patients in our series are listed in Table 1. Age, gender, primary tumor location and primary T classification did not differ significantly between recurrent and non-recurrent groups. However, neck N classification and previous neck treatment history differed significantly between the two groups.

**Table 1:** The clinical characteristics of the head and neck cancer patients in whom the treated necks were followed by US.

	Non-recurrent group (n = 85)	Recurrent group (n = 68)	P value
Age (years)	52.60 ± 10.93	52.49 ± 8.39	0.94
Gender			
Male	72/85 (84.71%)	62/68 (91.18%)	0.32
Female	13/85 (15.29%)	6/68 (8.82%)	
Primary tumor location			
Oral cavity	36/85 (42.35%)	30/68 (44.12%)	0.22
Nasopharynx	24/85 (28.24%)	15/68 (22.06%)	
Oropharynx/Hypopharynx/Larynx	19/85 (22.35%)	22/68 (32.35%)	
Others	6/85 (7.06%)	1/68 (1.47%)	
Primary T classification			
T1, T2	60/85 (70.59%)	47/68 (69.12%)	0.86
T3, T4	25/85 (29.41%)	21/68 (30.88%)	
Neck N classification			
N0	45/85 (52.9%)	16/68 (23.5%)	<0.001*
N + (N1,2,3)	40/85 (47.1%)	52/68 (76.5%)	
Previous neck treatment history			
Radiation alone	39/85 (45.88%)	28/68 (41.18%)	0.02
Neck dissection alone	33/85 (38.82%)	17/68 (25%)	
Radiation+Neck dissection	13/85 (15.29%)	23/68 (33.82%)	

Abbreviation: US, ultrasound; *using Fisher’s exact test.

In our series, 104 patients received US-guided FNA, 25 patients received US-guided CNB, and 24 patients received both US-guided FNA and CNB simultaneously. Of the 85 patients without LN recurrence, 16 patients (16/85, 18.82%) ever received open excisional biopsy after suspicion from the US examination, 69 (69/85, 81.18%) was confirmed at the 6-month follow-up appointment. Of the 68 patients with LN recurrence, 66 patients (66/68, 97.06%) received salvage neck surgery with final pathological verification. There were 2 recurrent patients misdiagnosed initially using US, and LN recurrences were confirmed months later. All the US features of the LNs are listed in Table 2. The US duration after previous treatment and target neck LN level were not significantly different between recurrent and non-recurrent groups. The period of time when the US started to demonstrate the signs of malignancy was during 2–115 months, with a mean of 17 ± 20 months after previous treatment. The target LN sizes (both short- and long-axis diameters) and US-guided tissue sampling methods differed significantly between the two groups. For suspicious US features, all the image characteristics differed significantly between the recurrent and non-recurrent groups.

**Table 2:** The US characteristics of the head and neck cancer patients in whom the treated necks were followed by US.

	Non-recurrent group (n = 85)	Recurrent group (n = 68)	P value
US duration after previous Tx (months)	17.35 ± 19.44	17.38 ± 22.08	0.99
Targeted neck LN level			
Upper neck (Level I, II)	57/85 (67.06%)	43/68 (63.24%)	0.73
Lower neck (Level III,IV,V)	28/85 (32.94%)	25/68 (36.76%)	
Targeted neck LN size, Long axis (cm)	1.40 ± 0.71	2.29 ± 1.18	<0.001
Targeted neck LN size, Short axis (cm)	0.73 ± 0.42	1.44 ± 0.94	<0.001
US-guided tissue sampling method			
FNA	75/85 (88.24%)	29/68 (42.65%)	<0.001
CNB	5/85 (5.88%)	20/68 (29.41%)	
FNA + CNB	5/85 (5.88%)	19/68 (27.94%)	
Suspicious US feature			
Size >0.8 cm (short axis diameter)	21/85 (24.71%)	54/68 (79.41%)	<0.001
Round shape, (S/L axis ratio >0.5)	35/85 (41.18%)	55/68 (80.88%)	<0.001
Hypoechogenicity	70/85 (82.35%)	64/68 (94.12%)	0.046*
Heterogeneous	18/85 (21.18%)	55/68 (80.88%)	<0.001
Irregular margin	16/85 (18.82%)	52/68 (76.47%)	<0.001
Different vascular pattern			<0.001
Chaotic pattern	1/85 (1.18%)	4/68 (5.88%)	
Linear hilum pattern	24/85 (28.24%)	1/68 (1.47%)	
Absent	60/85 (70.59%)	63/68 (92.64%)	

Abbreviation: US, ultrasound; LN, lymph node; Tx, treatment; FNA, fine needle aspiration; CNB, core needle biopsy; S/L axis ratio, short to long axis ratio; *using Fisher’s exact test.

### The sensitivity, specificity, positive predictive value and negative predictive value of different US-specific features and US-guided tissue sampling methods

The overall sensitivity, specificity, positive predictive value (PPV) and negative predictive value (NPV) of US-guided tissue sampling (FNA + CNB) in our series were 95.45%, 98.25%, 98.44% and 94.92%, respectively. Of the 128 cytological results from FNA, 38 patients (38/128, 29.69%) had non-diagnostic findings. Of the 49 pathological results from CNB, no patients had non-diagnostic findings. There were 23 tissue samples (23/49, 46.94%) from CNB that presented with fibrotic changes. Of these 23 CNB patients with fibrotic tissue, 6 patients (6/23, 26.09%) were initially misdiagnosed with non-recurrence. The sensitivity, specificity, PPV and NPV of all the suspicious US features and US-guided tissue samples are listed in Table 3. The specificities of hypoechogenicity and chaotic/absent vascular pattern were as low as 17.65% and 28.23%, respectively. Therefore, we further calculated the diagnostic accuracy of ≥2 suspected US features, excluding hypoechogenicity and chaotic/absent vascular pattern.

**Table 3:** Comparison of different US features and tissue sampling methods.

	PPV	NPV	Sensitivity	Specificity
Suspected US features				
Hypoechogenicity	47.76%	78.95%	94.12%	17.65%
Size >0.8 cm, short axis	72.0%	82.05%	79.41%	75.29%
Heterogeneous	75.34%	83.75%	80.88%	78.82%
Irregular margin	76.47%	81.18%	76.47%	81.18%
Chaotic or absent vascularity	52.34%	96.0%	98.53%	28.23%
Round shape (S/L axis ratio >0.5)	61.11%	79.37%	80.88%	58.82%
≥2 suspected US features (except hypoechogenicity and vascularity)	72.73%	93.85%	94.12%	71.76%
US-guided tissue sampling methods				
FNA	97.56%	95.92%	95.24%	97.92%
CNB	100%	62.5%	84.62%	100%
FNA + CNB	98.44%	94.92%	95.45%	98.25%

*Abbreviation: US, ultrasound; FNA, fine needle aspiration; CNB, core needle biopsy; PPV, positive predictive values; NPV, negative predictive values; S/L axis ratio, short/long axis ratio*.

Finally, all of the suspicious US features with significant differences between recurrent and non-recurrent patients (verified by Fisher’s exact tests or Chi-square tests) were further examined by multivariate logistic regression analysis (Table 4). This analysis revealed that the LN size (short-axis diameter >0.8 cm) (odds ratio (OR) 4.19, 95% confidence interval (CI) 1.47~11.95, p = 0.007), round shape (short/long axis ratio >0.5) (OR 3.44, 95% CI 1.12~10.54, p = 0.03), heterogeneous internal echo (OR 3.92, 95% CI 1.4~10.97, p = 0.009) and irregular margin (OR 7.32, 95% CI 2.55~21.02, p < 0.001) were still independent suspicious US features with significant differentiation. However, the hypoechogenicity (OR 2.38, 95% CI 0.48~11.83, p = 0.289) and chaotic/absent vascular pattern (OR 3.04, 95% CI 0.33~28.42, p = 0.330) were non-significant features in independently predicting recurrent LNs in our multivariate logistic regression model.

**Table 4:** Multivariate Logistic Regression Analyses of US features to predict the neck LN recurrence in treated head and neck cancer patients.

US features	OR	95% CI	P value
Size, LN short axis >0.8 cm	4.19	1.47~11.95	0.007
Round Shape (S/L axis ratio >0.5)	3.44	1.12~10.54	0.030
Heterogeneous	3.92	1.40~10.97	0.009
Chaotic or absent vascular pattern	3.04	0.33~28.42	0.330
Irregular margin	7.32	2.55~21.02	<0.001
Hypoechogenicity internal echo	2.38	0.48~11.83	0.289

*Abbreviation: US, ultrasound; LN, lymph node; S/L axis ratio, short/long axis ratio*.

## Discussion

For patients with head and neck cancer, early detection of the presence of LN recurrence in the treated neck during the follow-up period is paramount. Early detection of LN recurrence can initiate salvage treatment, either surgical intervention or radiotherapy, thereby having positive effects on the survival outcome for these patients^2^. However, as the morbidities of salvage treatment for neck LN recurrence are frequently significant, the diagnoses of LN recurrence should best be based on histologic verification. Nevertheless, surgical biopsy is unfavorable for the treated neck compared to the untreated neck because the scarred and fibrotic tissues after previous neck dissection and radiation are associated with an enhanced risk of complications such as large vessel injuries and poor wound healing. Therefore, one diagnostic tool with high accuracy and that allows ancillary tissue sampling for verification is ideal for this kind of situation. In general practice, we routinely use neck palpation and imaging examinations such as CT, MRI or PET to regularly check the treated neck. With the previous treatment-associated morbidities such as fibrosis or anatomic changes, the reported sensitivity and specificity for neck palpation to detect LN recurrence were not very reliable^15, 16^. When considering an imaging examination such as CT or MRI, the criteria of suspected LN recurrence mainly rely on the morphology of LNs such as size, shape, margin or necrosis^17^. In addition, a PET scan can provide additional information regarding tumor metabolism and therefore is more frequently used nowadays^18^. In clinical practice, we frequently prefer to observe the serial changes of physical and image examinations rather than to use single episode of the fitting criteria. However, this wait-and-see policy without ancillary tissue proof sometimes carries the immediate risk of delayed diagnoses. Moreover, some conditions, such as local infection or inflammation of the neck, could mimic LN recurrence in CT, MRI or PET examination^19^. Therefore, high false positive rates in imaging examinations are problems that frequently need to be faced in routine practice^20, 21^. All difficulties mentioned above can limit the early detection of LN recurrence in the treated neck of patients with head and neck cancer.

**Figure 1:**
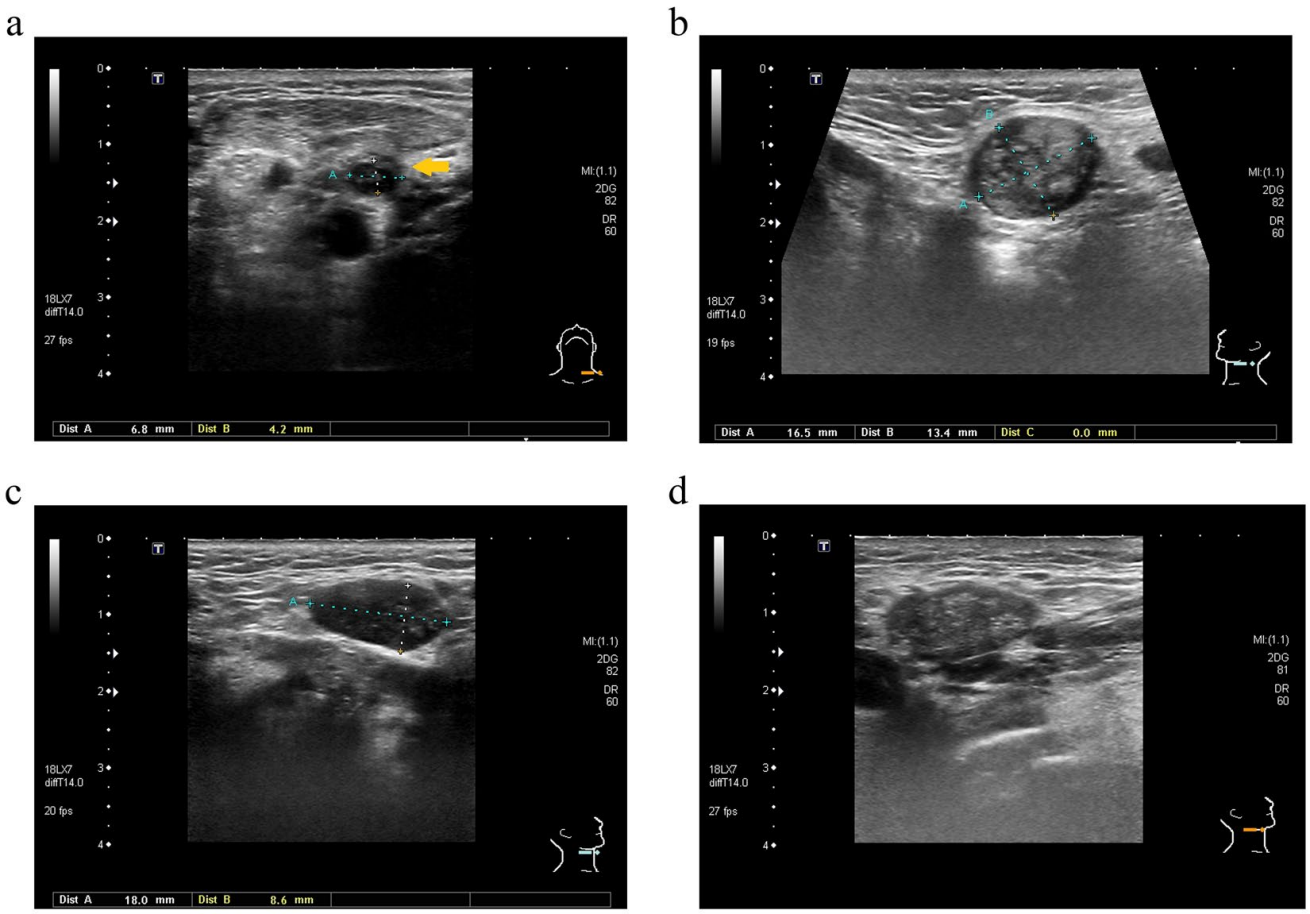
All significant ultrasound features of malignant lymph nodes (LNs) in the treated neck of head and neck cancer patients: (**a**) Left level IV recurrent LN with irregular margin (interrupted margin) (arrow), malignant (**b**) Left level IIa recurrent LN with round shape (short/long axis ratio >0.5), malignant (**c**) Right level Ib recurrent LN with size >0.8 cm, short axis, malignant (**d**) Right level Ib recurrent LN with heterogeneous internal echo, malignant.

**Figure 2:**
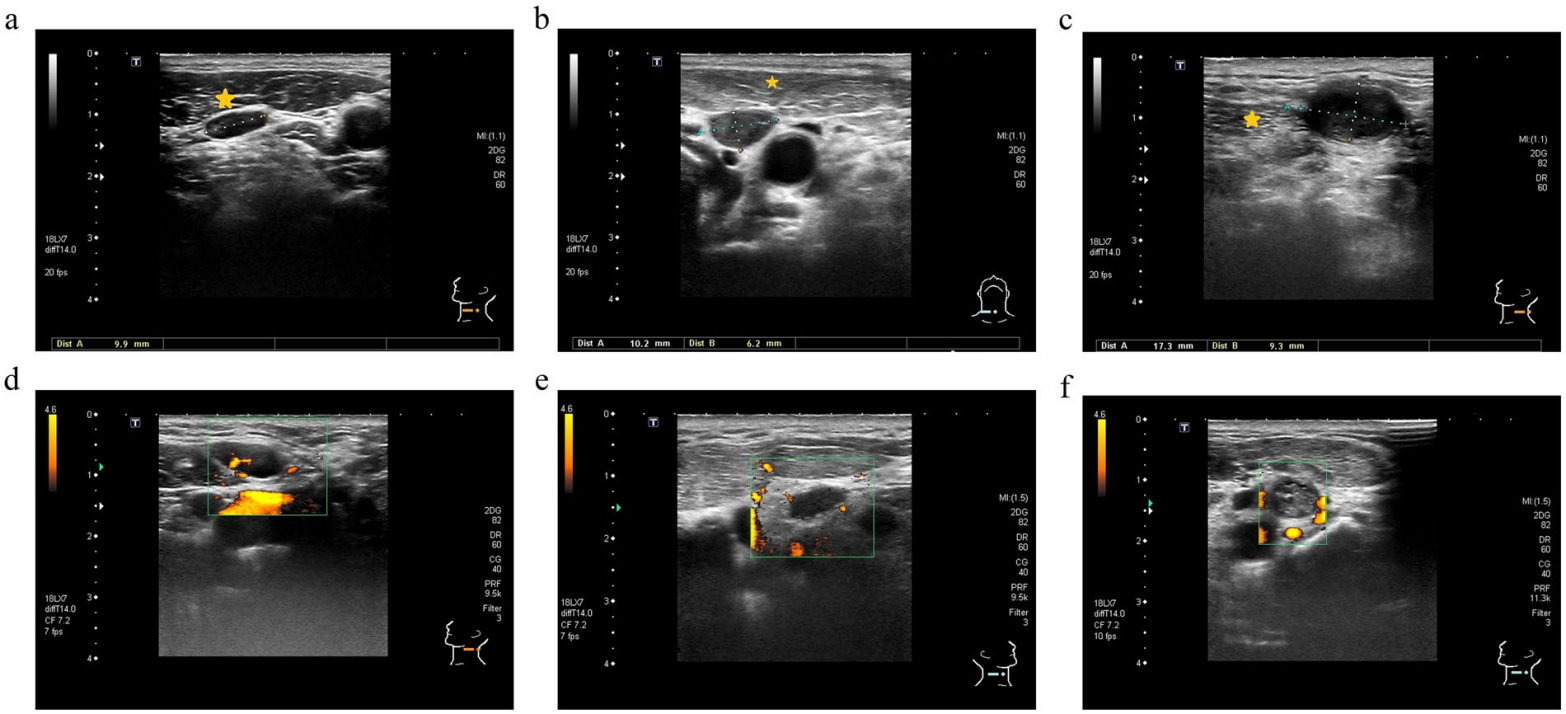
In comparison to the untreated neck, there are two ultrasound features of lymph nodes (hypoechogenicity and avascular pattern) that are more frequently noted in the treated neck: (**a**) Left level III benign LN in the untreated neck, isoechogenicity (equal to left sternocleidomastoid muscle (star) without fibrotic change) (**b**) Right level IV non-recurrent LN in the treated neck, hypoechogenicity (lower than right sternocleidomastoid muscle (star) with fibrotic change after previous neck dissection) (**c**) Left level Va recurrent LN in the treated neck, hypoechogenicity (lower than left sternocleidomastoid muscle (star) with fibrotic change after radiation) (**d**) Left level IIa benign LN in the untreated neck with typical linear hilum using a Doppler scan (**e**) Right level III non-recurrent LN in the treated neck, avascular pattern using a Doppler scan (**f**) Left level III recurrent LN in the treated neck, avascular pattern using a Doppler scan.

Compared to imaging examinations such as CT, MRI, and PET, US examination has several advantages, including low cost, low exposure to radiation and high acceptability to the patients. Most importantly, US can provide repeated examination and immediate ancillary US-guided tissue sampling if there are any suspicious LNs. Previously, for the untreated neck, several suspicious US features, including size (short-axis diameter >0.8 cm), round shape (short/long axis ratio >0.5), heterogeneous type of internal echo, hypoechogenicity, irregular margin, and chaotic/absent vascular pattern, have been reported to be highly associated with LN metastasis^8–14^. However, from our series, the results showed that the features of size (short-axis diameter >0.8 cm), round shape (short/long axis ratio >0.5), heterogeneous type of internal echo and irregular margin were still significant suspicious US features for the prediction of recurrent LNs in the treated neck (Fig. 1). In contrast, the other 2 suspicious US features, hypoechogenicity and chaotic/absent vascular pattern, which were reported to predict malignant LNs in the untreated neck, did not have similar abilities to differentiate recurrent LNs in the treated neck. In our opinion, these changes may result from the treatment-associated effects in the treated neck. First, previous neck dissection and/or radiation can result in significant fibrotic changes. These fibrotic changes will make the surrounding LN tissues present as more hyperechoic on US than in the untreated neck^22^. Therefore, the benign isoechogenic or hyperechogenic LNs comparatively show hypoechogenicity in the treated neck. In our series, 82.35% of non-recurrent LNs in the treated neck also showed hypoechogenicity using US examination (Fig. 2a,b and c). Therefore, hypoechogenicity may not be effective in predicting LN recurrence in the treated neck. Second, the fibrotic changes in the treated neck may result in the decrease of blood perfusion to the hilum or LN capsule generally and the LNs consequently show avascular patterns using the Doppler mode of US in the treated neck. In our series, 70.59% of non-recurrent LNs in the treated neck also showed avascular patterns using the Doppler mode of US, and only 5.88% of recurrent LNs in the treated neck showed chaotic vascular patterns. Therefore, the feature of chaotic/absent vascular pattern also may not be effective in predicting the recurrent LNs in the treated neck (Fig. 2d,e and f). However, even without these 2 suspicious US features, the combination of other suspicious US features (≥2 features, exclude hypoechogenicity and chaotic/absent vascular pattern) could still achieve acceptable sensitivity, specificity, PPV and NPV. Therefore, during US follow-up of the treated neck, the LNs with suspicious US features, including size >0.8 cm (short-axis), round shape (short/long axis ratio >0.5), irregular margin and heterogeneous internal echo still need to be seriously considered, and US-guided tissue sampling should be suggested if possible.

For the clinical characteristics of the treated head and neck cancer patients in our series, positive N stage was the only significant risk factor to predict recurrent neck LNs. Abundant studies have shown that the size and number of malignant LNs (N stage) are associated with neck regional recurrence^23^. Consequently, according to the treatment guidelines, neck dissection plus radiation are frequently advocated for patients with positive N stage. Therefore, in the recurrent group in our series, there were significantly more patients having received neck dissection plus radiation than in the non-recurrent group (Table 1). For primary tumor location and T stage, to the best of our knowledge, there are no convincing reports to clarify their relationships to neck nodal recurrence.

**Figure 3:**
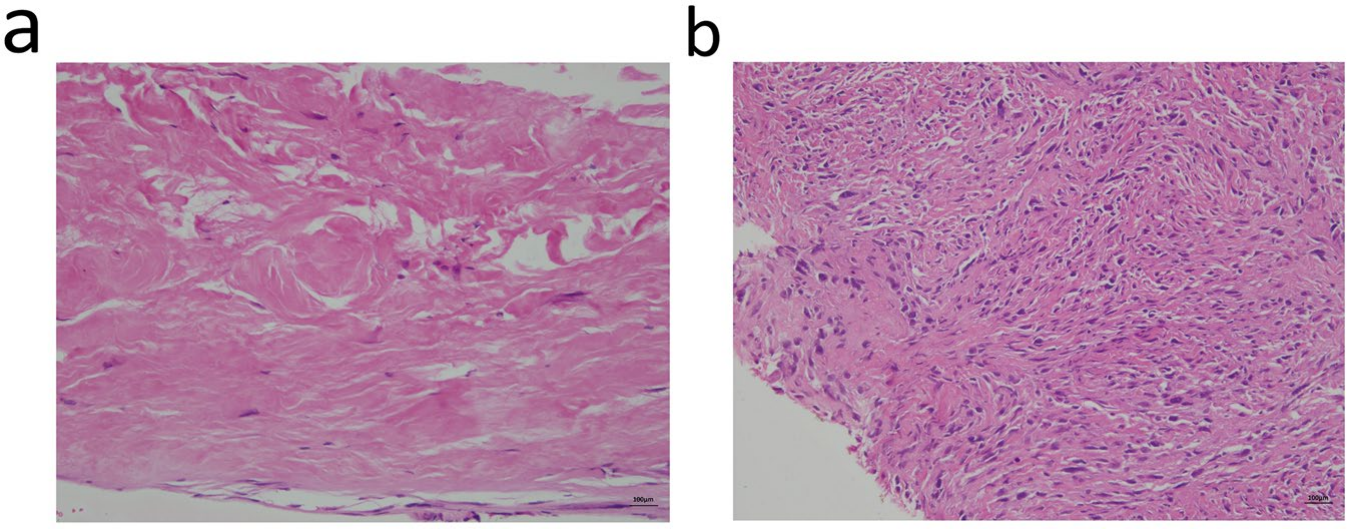
Pathologic pictures of fibrotic tissues in US-guided CNB samples in treated necks: (**a**) Left level IIa non-recurrent LN in the treated neck (benign fibro-adipose tissue with increased collagen deposition, hematoxylin and eosin stain, 200x) (**b**) Right level IV recurrent LN in the treated neck (malignant spindle cells in the fibrotic stroma, hematoxylin and eosin stain, 200x).

Regarding US-guided tissue sampling, there are two different methods used in clinical practice, namely FNA and CNB. It has been reported that in comparison to FNA, CNB has a higher diagnostic rate in detecting malignancy (99% vs. 90%)^24^. However, in our series, 6 patients (6/49, 12.24%) whose CNB tissues were initially mistaken as benign fibrotic tissues later received a final diagnosis of LN recurrence, which consequently led to a low NPV of CNB of 62.5%. In the untreated neck, the NPV of CNB is as high as approximately 93%^12^. In our opinion, the significantly low NPV in the treated neck may partially relate to the high incidence and proportion of fibrotic tissue presented in the US-guided CNB sample. In our series, nearly half (23/49, 46.94%) of the tissues from the US-guided CNB showed the presence of fibrotic tissue (Fig. 3). Therefore, it is important to emphasize that even if the CNB tissue report just shows fibrotic tissue, the surgical excisional biopsy or re-CNB should still be suggested for the highly suspicious LNs. For the US-guided FNA, our results showed that approximately one third of patients (38/128, 29.69%) did not have sufficient materials to establish the diagnoses. In the untreated neck, the non-diagnostic rate of FNA is only approximately 10%^25^. High non-diagnostic rates of FNA in the treated neck may be the reasons why in our series, the clinicians had more wills to perform CNB in the recurrent group (CNB: 29.41%, FNA: 42.65%) than in the non-recurrent group (CNB: 5.88%, FNA: 88.24%) (p < 0.001) (Table 2). This high non-diagnostic rate may be partially explained by the dense post-radiation fibrosis, which could impede the repeated “in and out” aspiration motion during the procedure. It could also be related to the absence of ancillary cytological examination immediately after US-guided FNA in our series. However, as the commercial CNB needle available for routine use has the minimal 1 cm cutting space, there are technical difficulties in performing CNB in small LNs less than 1 cm (long axis)^14, 24^. Therefore, FNA should still be the reasonable choice for targeted LNs smaller than 1 cm (long axis). However, if the FNA cytology could not provide the definite answer, repeated FNA, repeated CNB or surgical excision should still be suggested for the highly suspicious LNs. Overall in our series, except for the low NPV of CNB and the high non-diagnostic rate of FNA, the diagnostic accuracy of US-guided FNA/CNB in the treated neck was almost the same as in the untreated neck^12, 25^.

In the literatures, there were few other researches evaluating the technique utility of US-guided FNA in the diagnosis of neck nodal recurrence. Chan *et al*.^26^ had reported a significant worse sensitivity (40%), accuracy (54%), and NPV (37%) of FNA in the irradiated head and neck cancer patients with non-diagnostic rate of 30%^26^. Similar to our series, high non-diagnostic rate of FNA in the irradiated head and neck cancer patients was also noted. Both Nishimura *et al*.^27^ and Fleischman *et al*.^28^ had reported the good efficiency of US (Nishimura *et al*., sensitivity 88.2%, specificity 66.1%) and US-guided FNA (Nishimura *et al*., sensitivity 71.4%, specificity 95.6%; Fleischman *et al*., sensitivity 80%, specificity 100%, PPV 100%, NPV 92.3%, accuracy 88%) in detecting recurrence for the irradiated head and neck cancer patients^27, 28^. Lo *et al*.^29^ had reported that radiation had some influence on US features of malignant LNs (smaller short- and long-axis diameters, more frequent irregular margin, and less frequent distinguishable calcification) but had little influence on the diagnostic accuracy of FNA (sensitivity 97.1%, specificity 83.3%, PPV 94.3%, PPV 90.9%, accuracy 93.5%) in the treated oral cancer patients with non-diagnostic rate of 4.2%^29^. This result was also similar to our studies except the non-diagnostic rate. For US-guided CNB, to the best of our knowledge, there was no previous report to describe its diagnostic accuracy in the treated head and neck cancer patients.

There were some limitations to our study. First, this is a retrospective study and thus it might contain various types of bias, like selection bias and information bias. As for selection bias, we excluded the patients who had obvious benign LN features in US and thus did not receive FNA or CNB. This may underestimate the NPV of US features. We also excluded the patients who had obvious malignant LN features in US (e.g. extremely large LN size >6 cm) but did not receive FNA or CNB because further neck dissection surgery was arranged directly. This may underestimate the PPV of US features. As for information bias, the final diagnosis of recurrence or not in our study was not totally based on pathologic verification. For the patients who had benign FNA cytology result and then did not receive further open biopsy or salvage neck dissection, if the neck LN remained stable, or became remitted 6 months later, we would also classify it as non-recurrence. This may result in grouping error and mislead the final results. Second, even though the US readings in our series were assessed by two senior sonographers who had US experience of more than 2000 cases each (C-P.W. and T-C.C.), its results could still have some degree of operator-dependent bias. Third, US waves cannot transmit through bone and air and subsequently could not visualize the LNs behind bones and air. Fourth, we did not have the service of ancillary cytological examination immediately after US-guided FNA in our hospital. Therefore, there are areas that require improvement regarding our FNA result. In conclusion, we suggest that US with ancillary US-guided FNA or CNB are effective diagnostic tools for neck nodal recurrence surveillance.

## Materials and Methods

### Patient population

We retrospectively reviewed the head and neck US reports of all patients with a history of cancers in the head and neck region (including oral cancer, oropharyngeal cancer, hypopharyngeal cancer, laryngeal cancer, nasopharyngeal carcinoma, salivary gland cancer, and unknown primary cancer) from January 2011 to December 2014 at National Taiwan University Hospital and National Taiwan University Hospital, Yun-Lin Branch. The Research Ethics Committee of the hospital approved the study (NTUH IRB-201608066RIND) and all methods were performed in accordance with the relevant guidelines and regulations. The exclusion criteria were patients with a history of cancer other than head and neck cancer, patients who received US examination without US-guided FNA and/or CNB, patients who received US examination without image records, and patients who did not have medical records of follow-up 6 months after US examination. Therefore, all of the patients included in our series had image records of US features combined with the cyto-histologic information acquired by means of US-guided FNA and/or CNB. Furthermore, all patients in our series received follow-up again 6 months later by routine survey using neck palpation, imaging CT, or MRI to exclude the misdiagnoses of US examination. The definite diagnoses of LN recurrence were based on the pathologic report from salvage surgery or open excisional biopsy. The TNM status of head and neck malignancies was classified according to the 2010 criteria of the American Joint Committee on Cancer (AJCC)^30^.

### The procedures of US examination and US-guided FNA and CNB

The head and neck US was performed (Toshiba Aplio SSA790 diagnostic US system, Tochigi-ken, Japan or Hitachi HI VISION Avius®, Soto-kanda, Chiyoda-ku, Tokyo, Japan) with a 12-MHz linear array transducer. After grossly surveying the bilateral neck from level I to level VI, all images with suspicious US features were recorded. Suspicious US features included size (short-axis diameter >0.8 cm), hypoechogenicity, heterogeneous type of internal echo, irregular margin, chaotic/absent vascular pattern and round shape (taller than wide, short/long axis ratio >0.5)^4–9^. After obtaining the informed consent of the patients, US-guided FNA and/or CNB were applied to the LNs with suspicious US features. If the long-axis diameters of targeted LNs were smaller than 1 cm, only US-guided FNA would be performed. If the long-axis diameters of suspected LNs were greater than 1 cm, US-guided FNA and/or CNB would be performed depending on the operator’s preference. The procedure of FNA was performed using the free-hand technique with a 22-gauge needle/20 ml syringe connected to a Cameco holder without local anesthesia as previously described^29^. The tip position of the fine needle was then confirmed to be located in the targeted LN by US. The procedure of CNB was performed using the free-hand technique with an 18-gauge core needle (Temno Evolution™ Biopsy Devices, Cardinal Health Inc., Dublin, CA, USA) under local anesthesia as previously described^31^. The FNA/CNB results were defined as “positive” if malignant or atypical cells were present; “negative” if no malignant cells were observed; and “non-diagnostic” if there were insufficient cells, obvious crushing injuries, or too many RBCs obscuring the cells.

### Statistical analysis

All statistical analyses were performed using the SPSS software package, version 16.0 (SPSS Inc., Chicago, IL, USA). Fisher’s exact tests and Chi-square tests were used to determine differences in the clinical and US feature between the patients with and without LN recurrence in the treated neck. The primary outcomes were the sensitivity, specificity, PPV and NPV of different US features in detecting LN recurrence. The secondary outcomes were the sensitivity, specificity, PPV and NPV of US-guided FNA and/or CNB in verification of LN recurrence. All potential US features were further analyzed using a multivariate logistic regression model. Corresponding p values < 0.05 were interpreted as statistically significant.

## References

[1] Wong LY, Wei WI, Lam LK, Yuen AP (2003). Salvage of recurrent head and neck squamous cell carcinoma after primary curative surgery. Head & Neck.

[2] Ho AS (2014). Decision making in the management of recurrent head and neck cancer. Head & Neck.

[3] Shaw SM (2016). Valid and reliable techniques for measuring fibrosis in patients with head and neck cancer postradiotherapy: A systematic review. Head & Neck.

[4] Liao LJ, Wang CT, Young YH, Cheng PW (2010). Real-time and computerized sonographic scoring system for predicting malignant cervical lymphadenopathy. Head & Neck.

[5] Gupta A (2011). Sonographic assessment of cervical lymphadenopathy: role of high-resolution and color Doppler imaging. Head & Neck.

[6] Chikui T, Yonetsu K, Nakamura T (2000). Multivariate feature analysis of sonographic findings of metastatic cervical lymph nodes: contribution of blood flow features revealed by power Doppler sonography for predicting metastasis. American Journal of Neuroradiology.

[7] Wu M, Chen H, Zheng X, Burstein DE (2013). Evaluation of a scoring system for predicting lymph node malignancy in ultrasound guided fine needle aspiration practice. Diagnostic Cytopathology..

[8] Ying M (2013). Review of ultrasonography of malignant neck nodes: greyscale, Doppler, contrast enhancement and elastography. Cancer Imaging.

[9] Lyshchik A (2007). Cervical lymph node metastases: diagnosis at sonoelastography initial experience. Radiology..

[10] de Bondt RB (2007). Detection of lymph node metastases in head and neck cancer: a meta-analysis comparing US, USgFNAC, CT and MR imaging. European Journal of Radiology.

[11] Screaton NJ, Berman LH, Grant JW (2002). Head and neck lymphadenopathy: evaluation with US-guided cutting-needle biopsy. Radiology..

[12] Novoa E, Gurtler N, Arnoux A, Kraft M (2012). Role of ultrasound-guided core-needle biopsy in the assessment of head and neck lesions: a meta-analysis and systematic review of the literature. Head & Neck.

[13] Saha S (2011). Ultrasound guided Core Biopsy, Fine Needle Aspiration Cytology and Surgical Excision Biopsy in the diagnosis of metastatic squamous cell carcinoma in the head and neck: an eleven year experience. European Journal of Radiology.

[14] Pfeiffer J (2007). Ultrasound-guided core-needle biopsy in the diagnosis of head and neck masses: indications, technique, and results. Head & Neck.

[15] Haberal I (2004). Which is important in the evaluation of metastatic lymph nodes in head and neck cancer: palpation, ultrasonography, or computed tomography?. Otolaryngology–Head and Neck Surgery.

[16] Baatenburg de Jong RJ (1989). Metastatic neck disease. Palpation vs ultrasound examination. Archives of Otolaryngology–Head & Neck Surgery.

[17] Hoang JK, Vanka J, Ludwig BJ, Glastonbury CM (2013). Evaluation of cervical lymph nodes in head and neck cancer with CT and MRI: tips, traps, and a systematic approach. American Journal of Roentgenology.

[18] Paidpally V (2012). FDG-PET/CT imaging biomarkers in head and neck squamous cell carcinoma. Imaging in Medicine.

[19] Purohit BS (2014). FDG-PET/CT pitfalls in oncological head and neck imaging. Insights into Imaging.

[20] Fogh SE (2012). Value of fluoro-2-deoxy-D-glucose-positron emission tomography for detecting metastatic lesions in head and neck cancer. American Journal of Clinical Oncology.

[21] de Bree R (2009). Detection of locoregional recurrent head and neck cancer after (chemo)radiotherapy using modern imaging. Oral Oncology..

[22] Yang X (2012). Ultrasound GLCM texture analysis of radiation-induced parotid-gland injury in head-and-neck cancer radiotherapy: an *in vivo* study of late toxicity. Medical Physics..

[23] Kim, S., Smith, B. D., Haffty, B. G. Chapter 3: Prognostic Factors in Patients with Head and Neck Cancer. *Head and Neck Cancer: A Multidisciplinary Approach* (4^th^ ed.) 88–112 (Wolters Kluwer Health/Lippincott Williams & Wilkins, 2014).

[24] Kraft M (2008). Comparison of ultrasound-guided core-needle biopsy and fine-needle aspiration in the assessment of head and neck lesions. Head & Neck.

[25] Goret CC (2015). Diagnostic value of fine needle aspiration biopsy in non-thyroidal head and neck lesions: a retrospective study of 866 aspiration materials. Int J Clin Exp Pathol.

[26] Chan RC-L, Chan J (2012). Y-W. Effect of previous radiotherapy on cervical lymph node fine-needle aspiration cytology diagnostic accuracy in head and neck cancers. Laryngoscope..

[27] Nishimura G (2012). Treatment evaluation of metastatic lymph nodes after concurrent chemoradiotherapy in patients with head and neck squamous cell carcinoma. Anticancer Res..

[28] Fleischman GM, Thorp BD, Difurio M, Hackman TG (2016). Accuracy of ultrasonography-guided fine-needle aspiration in detecting persistent nodal disease after chemoradiotherapy. JAMA Otolaryngol Head Neck Surg.

[29] Lo WC (2016). The Effect of Radiotherapy on Ultrasound-Guided Fine Needle Aspiration Biopsy and the Ultrasound Characteristics of Neck Lymph Nodes in Oral Cancer Patients after Primary Treatment. PLoS One.

[30] Edge, S. American Joint Committee on Cancer (AJCC) Cancer staging handbook: from the AJCC cancer staging manual (19^th^ ed.) 718 (Springer, 2010).

[31] Chen CN (2016). Application of Ultrasound-Guided Core Biopsy to Minimal-Invasively Diagnose Supraclavicular Fossa Tumors and Minimize the Requirement of Invasive Diagnostic Surgery. Medicine..

